# Circadian Rhythm Dysregulation and Restoration: The Role of Melatonin

**DOI:** 10.3390/nu13103480

**Published:** 2021-09-30

**Authors:** Clayton Vasey, Jennifer McBride, Kayla Penta

**Affiliations:** 1 School of Pharmacy, College of Medicine, Lake Erie College of Osteopathic Medicine, Erie, PA 16509, USA; 2 COVID/Communicable Disease, Cabarrus Health Alliance, Kannapolis, NC 28081, USA; Jennifer.mcbride@cabarrushealth.org; 3 College of Medicine, Lake Erie College of Osteopathic Medicine, Elmira, NY 14901, USA; kpenta@lecom.edu

**Keywords:** melatonin, sleep, circadian rhythm, bright light therapy, delayed sleep phase disorder

## Abstract

Sleep is an essential component of overall human health but is so tightly regulated that when disrupted can cause or worsen certain ailments. An important part of this process is the presence of the well-known hormone, melatonin. This compound assists in the governing of sleep and circadian rhythms. Previous studies have postulated that dysregulation of melatonin rhythms is the driving force behind sleep and circadian disorders. A computer-aided search spanning the years of 2015–2020 using the search terms melatonin, circadian rhythm, disorder yielded 52 full text articles that were analyzed. We explored the mechanisms behind melatonin dysregulation and how it affects various disorders. Additionally, we examined associated therapeutic treatments including bright light therapy (BLT) and exogenous forms of melatonin. We found that over the past 5 years, melatonin has not been widely investigated in clinical studies thus there remains large gaps in its potential utilization as a therapy.

## 1. Introduction

Sleep is an essential component of overall good human health. It is a state of consciousness that occurs in a series of stages where the senses are largely ignored, motor function is mostly inhibited, and dreaming can occur [1]. These stages include Stages I to IV and Rapid Eye Movement (REM). The necessary or proper quantity and quality of sleep maintains brain, heart, and immune health [2] as well as aiding in memory consolidation and restoration of brain function [3].

Humans normally experience sleep cyclically once a day due to a tightly regulated circadian control. Depending on age, the recommended duration of sleep differs. For example, adolescents should sleep for 9 h per night while adults only need 7–8 h [1]. Neuronal, neurotransmitter, hormonal, and genetic factors regulate the propensity to sleep, notably adenosine, serotonin, and melatonin. Adenosine is an endogenous purine nucleoside with a variety of physiologic functions, including the promotion of sleep [4]. Serotonin is a neurotransmitter that plays a role in mood, sleep, and emotion [5]. Melatonin is an indole hormone derived from serotonin via the tryptophan-serotonin biosynthetic pathway in the pineal gland in varying concentrations based on input from circadian centers of the brain [6]. Melatonin is also involved with the promotion of sleep, timing of other circadian functions, immune regulation, and modulation of pituitary and adrenal hormones [6]. In this review, we will be focusing specifically on melatonin.

To promote sleep, melatonin concentrations rise as light fades prior to darkness, peak during darkness, and fall when exposed to light to promote wakefulness [6]. Although endogenous in most, some individuals may be deficient in or lack the ability to appropriately release melatonin where melatonin supplementation is an option. Melatonin supplements are relatively safe, non-habit forming, and have low potential for abuse [7]. Disruption of sleep and sudden changes in sleep cycles causes melatonin release to become out of sync with environmental cues which have been associated with loss of concentration and cognitive disease, susceptibility to cardiovascular and metabolic disease, and a weakened immune system [2,3]. 

Melatonin production is suppressed when light is detected; therefore, increased light exposure leads to decreased circulating melatonin in the blood plasma. Shift workers and those that live in proximity to the Earth’s poles are exposed to abnormal patterns of light and should then, in theory, develop aberrant melatonin rhythms.

In this review, we investigate the gaps in knowledge regarding melatonin’s role in the creation and dysregulation of circadian rhythms, in individuals with and without linked comorbidities. We also examine melatonin’s potential therapeutic use in restoring circadian rhythms in those individuals experiencing sleep and circadian pathologies. The use and sale of melatonin as a supplement has reportedly grown approximately 29% from 2018 to 2019 [8]. During this timeframe, we felt as if there were few clinical data driving this increase in use; therefore, we aimed to investigate melatonin for potential clinical use.

## 2. Materials and Methods

A systematic literature search was conducted on peer-reviewed scientific journal articles. As suggested by Brocke et al. the literature review shall (1) define the scope of the review, (2) conceptualize the topic, (3) conduct a literature search, (4) analyze and synthesize the found literature, and (5) plan a research agenda. The first four steps are applied in the review, but the fifth one is not, as the intention of this literature review was not to find research gaps but to analyze the existing scientific evidence on the light-induced impacts on circadian rhythm in humans [9].

### Concepts and Inclusion Criteria

**Figure 1:**
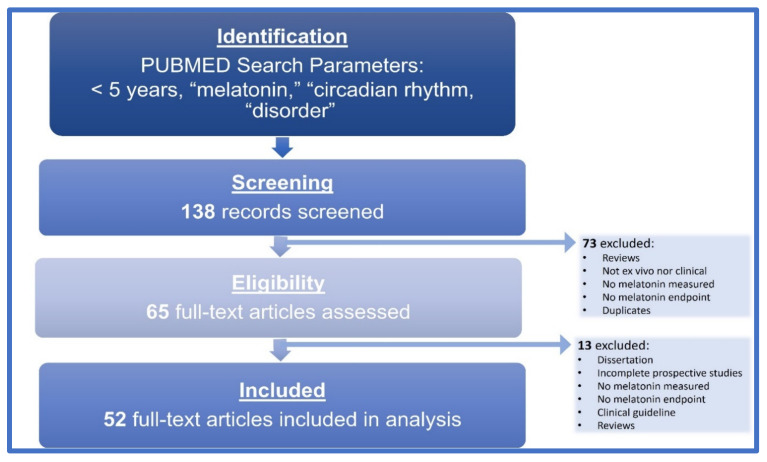
Literature selection flow chart. All steps of the literature search are represented within the flow chart, resulting in 52 full-text articles included in this analysis.

We performed a computer-aided search of PubMed from 2015–2020 using the following search terms: melatonin, circadian rhythm, disorder. The search was conducted on 21 December 2020. The papers were deemed relevant on the basis of their title, abstract and finally full text, resulting in 138 relevant papers. All authors screened all potentially relevant publications. Studies were initially included if they (1) were scientific papers, (2) papers presenting original experimental studies (3) written in the English language (4) measured melatonin as a study endpoint. The quality criteria resulted in 52 qualified papers, represented in Figure 1.

## 3. Background

### 3.1. Daylight Hours

Understanding normal physiology of sleep is imperative to understanding dysfunctional sleep and circadian rhythms. Sleep is a necessary and useful physiological process in humans, controlled by circadian rhythms. These circadian rhythms are daily biological cycles that control a large portion of physiological processes [10]. Both natural and artificial light heavily influences circadian rhythms by entraining “endogenous oscillators” that are composed of neural, hormonal, and genetic elements [11]. There are central and peripheral oscillators that play roles in specific parts of the circadian system. The major central oscillator is the suprachiasmatic nucleus (SCN); a paired nucleus in the hypothalamus of humans and other mammals that receives input from specific neurons in the retina [12]. Peripheral oscillators are controlled by the rhythm of the SCN. The rhythm of the SCN is generated by the cyclical expression of clock genes in the 20,000 neurons that compose it [13]. Clock genes encode transcription factors called clock proteins, “whose levels rise and fall in a regular cyclic or oscillating pattern” [13]. This leads to specific processes occurring at specific times. 

**Figure 2:**
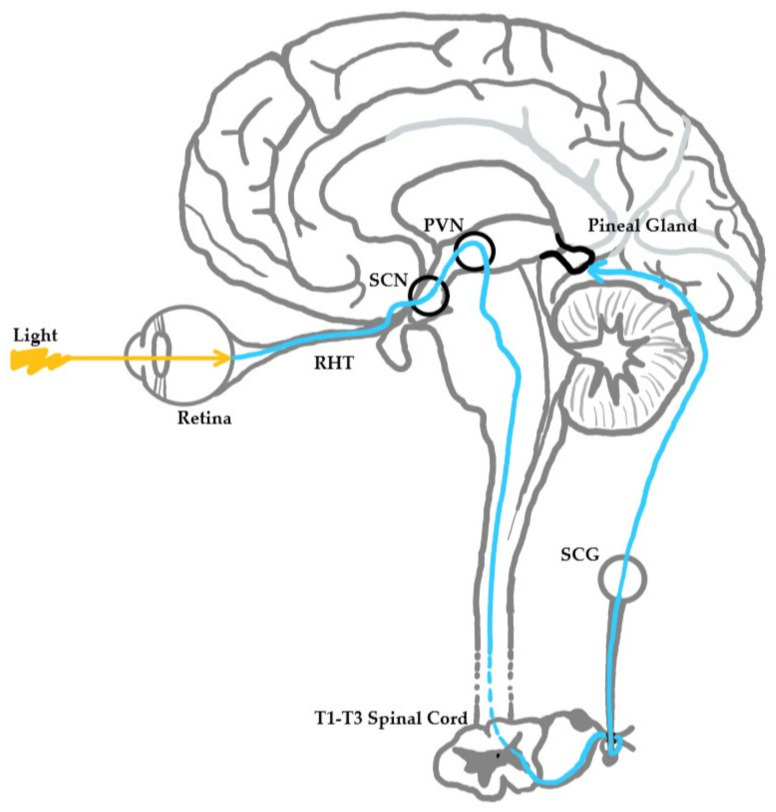
Neuroanatomical pathway of light stimulus to the pineal gland. Light strikes the retina, which results in a neuronal signaling cascade from retina to retinohypothalamic tract (RHT) to suprachiasmatic nucleus (SCN) to paraventricular nucleus (PVN) to the brainstem to the spinal cord (levels T1-T3) to the superior cervical ganglion (SCG) to the pineal gland.

The process starts when the SCN stimulates the pineal gland, an effector of these physiological processes, through a neural pathway. This pathway begins with the SCN signaling the medial forebrain bundle through descending hypothalamic projections. The medial forebrain bundle then projects to the spinal cord, which in turn projects to the superior cervical ganglia. The superior cervical ganglia then provide sympathetic innervation to the pineal gland [11]. Light influences this pathway when it is detected by specific retinal cells called intrinsically photosensitive retinal ganglion cells (ipRGCs) that send light intensity and wavelength information to the SCN by way of the retinohypothalamic tract. Through this pathway, light information reaches the pineal gland, where melatonin metabolism is controlled (Figure 2). 

Melatonin is known to play a role in promoting a sleep state. Therefore, absent or reduced melatonin levels should perpetuate an awake state. It is already known that exposure to light, especially high wavelength light, suppresses circulating melatonin in the blood [11]. Suppression of plasma melatonin is thought to result from the inhibition of melatonin synthetic enzymes like N-acetyltransferase in the pineal gland that are under the influence of external light [11]. Normally, this process inhibits sleepiness during the day when diurnal humans need to be active. However, exposure to high wavelength light during abnormal times disrupts circadian rhythms and inappropriately suppresses melatonin synthesis and secretion in the pineal gland. Numerous studies have shown chronic and episodic suppression of melatonin to be potentially pathogenic and carcinogenic, which are discussed later in this review [14,15,16,17]. 

There are several examples of abnormal light exposures that humans regularly experience. One of which is residence near the Earth’s poles, where light or darkness can last for weeks at a time. Those residing nearest the Earth’s poles experience weeks at a time where there is total darkness or unrelenting sunshine. At Halley, Antarctica, individuals experience 110 consecutive days of darkness in the winter and 110 consecutive days of light in the summer [18]. Because of these light conditions, humans have been documented to experience decreases in slow-wave sleep (SWS), increases in stage R sleep, and sleep fragmentation [19]. Researchers living there complain of poor sleep; however, a team found that phototherapy involving exposure to standard white and blue-enriched light throughout the subjective day remedied sleep timing delay [18]. In addition to phototherapy, exogenous melatonin supplementation at appropriate times was shown to be effective in improving subjective, but not objective sleep parameters [17]. Appropriate light exposure during the extended period of diminished light corrected the aberrant melatonin rhythm in these individuals.

Rotating or night shift work is a subset of modern nocturnal artificial light that exposes workers to light at inappropriate times. Studies have shown that nocturnal light causes desynchrony of cortisol, body temperature, and melatonin rhythms [20] as well as decreased melatonin production [21] in night shift nurses. There is conflicting evidence on whether or not the phase of circadian clock genes can be delayed by shift work. One team found that certain clock gene phases can be delayed, while other clock gene phases are unchanged by shift work [22]. Other researchers have found no significant delay in clock gene phase attributable to shift work [20]. Discrepancies may be due to a number of criteria, including but not limited to differences in length of the study, length of the night shift work, and differences in measurement methods. Regardless of the inconsistent findings, in 2019, the International Agency for Research on Cancer classified night shift work as a probable carcinogen to humans (Group 2A) [22].

Finally, increased global ambient light and light-emitting technology use is another common incident of abnormal light exposure. Light-emitting technology includes smartphones, tablets, computers, and televisions. Increasing ambient light and light-emitting technology use exposes humans to light at inappropriate times. Following the installation of the first electrical lighting system in New York in 1882, the world has followed suit and greatly expanded the amount of light in the environment to a point where humans live outside of natural light-dark cycles [23]. Some have termed this luminous infiltration as light pollution or light toxicity. As early as 1980, light at night (LAN) from artificial sources was known to be an environmental pollutant [23]. As LAN research continued, many studies concluded that LAN is involved in suppression of pineal melatonin production [23,24]. Additionally, light has since permeated the darkness in a more individualized way. In a 2015 study, 90% of Americans were found to use light-emitting electronic devices at least several nights a week prior to sleep, further contributing to disruptions in melatonin rhythm [25].

Many studies have shown that the wavelength of light is an important factor in suppression of melatonin. Specifically, the human circadian pacemaker in the SCN is more sensitive to short or blue wavelength light (460 nm) than to long or red wavelength light (555 nm), as the short wavelength has been shown to suppress melatonin [24,25,26]. Intensity of the light also has a negative effect on sleep, but of lesser magnitude than wavelength of light [27]. Alternatively, limiting short-wavelength light exposure has been shown to significantly shorten sleep onset latency by 7 min, improve sleep quality, and increase alertness the following morning [28]. In 2018, melatonin was also investigated as a potential therapy for intensive care unit (ICU) patients experiencing symptoms of insomnia in a study that did not yield significant results [29]. Appropriate light exposure corrected aberrant endogenous melatonin rhythm; however, exogenous melatonin is unpredictable in its capacity to restore normal sleep.

### 3.2. Endogenous Melatonin

**Figure 3:**
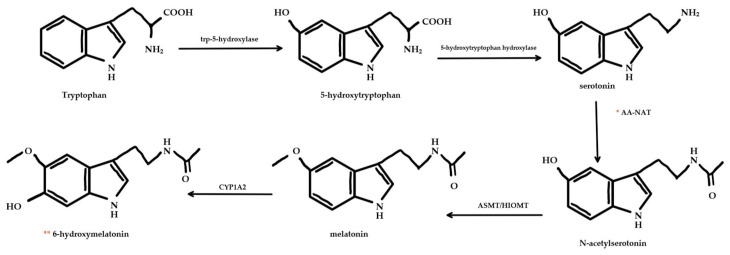
Melatonin metabolic pathway. Melatonin is synthesized from tryptophan and serotonin by a series of enzymes. * AA-NAT enzyme is the rate-limiting enzyme of this pathway. Melatonin is catabolized by cytochrome P450 enzymes (like CYP1A2) into 6-hydroxymelatonin. ** 6-hydroxymelatonin can be excreted or sulfated and then excreted.

Endogenous melatonin is synthesized in the pinealocyte, and other tissues, where it is an end-product of tryptophan and serotonin biosynthetic pathways (Figure 3). Initially, tryptophan is transported into the cell, where it is acted on by tryptophan-5-hydroxylase and 5-hydroxytryptophan decarboxylase enzymes to form serotonin [30]. Depending on adrenergic neuronal input, serotonin is acetylated by arylalkylamine-N-acetyltransferase (AA-NAT) then methylated by “acetylserotonin-O-methyltransferase (ASMT, also called hydroxyindole-O-methyltransferase or HIOMT)” to form melatonin [30]. AA-NAT is the rate-limiting enzyme in the synthesis of melatonin [30]. As such, it is a likely site of regulation in the synthesis of melatonin. 

**Figure 4:**
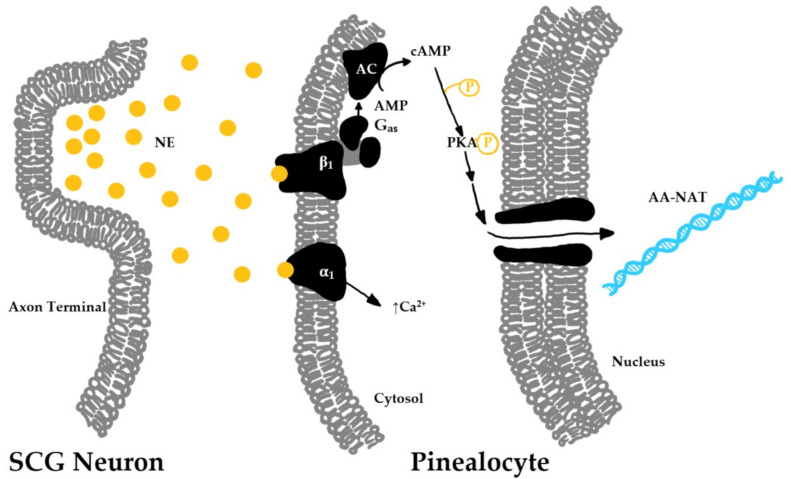
Transcription pathway for melatonin biosynthetic enzymes. The axon terminal of the neuron originating from the superior cervical ganglion releases norepinephrine (NE) into the synapse when stimulated. NE binds to beta-1 (β1) and alpha-1 (α1) receptors on the cell membrane of pineal gland cells (pinealocytes). β1 receptor stimulation leads to activation of downstream signaling of Gαs, adenylate cyclase (AC), and phosphorylated protein kinase A (PKA-P) to stimulate the transcription of AA-NAT RNA which will be transcribed into biosynthetic enzymes to synthesize melatonin.

Signal transduction in the pinealocyte begins in the membrane, where adrenergic neurons projecting from the superior cervical ganglion release norepinephrine to act on β1- and α1-adrenergic receptors (Figure 4) [31]. β1-adrenergic receptors, when stimulated by norepinephrine, signal adenylate cyclase to increase cytoplasmic cAMP, which leads to an activation of cAMP-dependent protein kinase A (PKA), which then acts through a signal cascade to stimulate production of AA-NAT. In addition, α1-adrenergic receptors are stimulated by norepinephrine, which leads to an increase in cytoplasmic calcium ions [32]. It is still unclear what this increase in calcium ion concentration accomplishes. It has been hypothesized that the calcium surge is important for diversifying the adrenergic signal transduction pathway to act on AA-NAT [33].

Regulation of endogenous melatonin release is incredibly complex with many moving parts. In summary, release is upregulated by light through the neural and cellular processes previously outlined above. Downregulation is mediated by a withdrawal of norepinephrine release from the neural pathway, decreased cytoplasmic secondary messengers, inhibition of the *aa-nat* gene by inhibitory elements, and increased degradation of cytoplasmic AA-NAT [33].

There are several variabilities in regard to endogenous melatonin regulation. In humans, many discrepancies exist concerning the release of hormones and other metabolic products between males and females. It has been hypothesized that melatonin rhythms may follow this pattern as well. However, studies have not been able to come to a consensus. There have been studies that have reported sex-based differences when in a highly regulated environment, however when left to self-selection of lighting times, these differences are no longer present [7,34,35,36]. Additionally, having a small sample size and inconsistent variables makes it difficult to compare results across studies. 

Another area of interest is how the aging process affects melatonin production. Studies have shown conflicting evidence that the level of melatonin released could be age dependent. Earlier studies from the 1980s have shown that peak melatonin levels decrease from age 1 to 18 [37] and continue to decrease in adults as they reach more advanced ages [38]. Recent evidence supports this concept, suggesting that decreases in peak melatonin release in older adults may be attributed to pathological processes [16]. The melatonin producing function of the pineal gland is diminished during the aging process and the gland is one of the most common sites in the adult body to undergo calcification [12].

Endogenous melatonin is not only found and produced in the pineal gland. Extra-pineal sources of melatonin can be found in most of the major systems of the body, most of which also contain melatonin biosynthetic enzymes [31]. Presence of these enzymes and melatonin implies the synthesis of melatonin in these tissues, and typically includes the presence of melatonin receptors.

### 3.3. Exogenous Melatonin

For those who experience difficulty falling asleep or staying asleep, oral melatonin supplementation has been considered for therapy, although its efficacy is uncertain. Andersen et al. found in a human pharmacokinetics study that 10 mg oral melatonin had an average bioavailability of only 3% due to extensive hepatic first pass metabolism [39]. This percentage varied widely amongst the healthy young male participants. A study published the following year found a mean bioavailability 33% with a range of 10% to 56% [30].

Oral melatonin, typically in tablet or capsule form, can be absorbed relatively rapidly. It is absorbed following first order, or concentration-dependent, kinetics [39]. First-order kinetic absorption of melatonin implies that absorption is not saturable, so larger doses should result in higher plasma concentrations. A pharmacokinetic study of oral and intravenous melatonin administration in healthy males found that melatonin pharmacokinetics vary greatly between individuals. These researchers found that oral melatonin reaches peak plasma concentration after 41 min, which fits into the previously established range of 30 to 60 min [39]. Therefore, data suggest that for maximal effect, oral melatonin should be taken approximately 40 min before attempting to sleep.

In the event that melatonin release is altered by pathologic processes or supplemented by exogenous sources, compensatory mechanisms for replenishment or decrease have not yet been identified. Neither tolerance, sensitization, nor habituation have been shown to develop. Further investigation is needed to determine if prolonged regular usage may result in the development of these effects. Animal studies done on diurnal primates showed that “5-mg/kg dose for 4 weeks or gradually escalating melatonin doses (5–320 mg/kg over a 3-week period) did not result in the development of tolerance or sensitization to the effect of melatonin on sleep initiation or sleep period” [40]. In 2013, a meta-analysis investigating the efficacy of melatonin supplements concluded that melatonin use precipitated little to no development of tolerance or habituation [41].

Melatonin is noted for its safety as a lethal dose has yet to be clinically established. In a 1967 study, mice survived administration of 800 mg/kg doses of melatonin with no significant adverse effects [42]. In fact, “melatonin has been given to humans for a 1-month period at a relatively high dose (1 g daily taken per os)” with researchers having observed high plasma concentrations of melatonin, headache, dizziness, and drowsiness without evidence of ocular toxicity, hepatoxicity, nephrotoxicity, or myelotoxicity [31]. A melatonin dose–response curve was not discovered during the research for this review, although these data would be beneficial. Safety has not yet been evaluated in pregnant women, and only to a limited extent in children. Physicians recommend that those with “epilepsy and those taking blood thinner medications need to be under medical supervision when taking melatonin supplements” [43].

### 3.4. Dysfunction and Disorders

Melatonin and its effects can be observed in most of the major systems of the body. Extra-pineal sources of melatonin include “brain, retina, lens, cochlea, Harderian gland, airway epithelium, skin, gastrointestinal tract, liver, kidney, thyroid, pancreas, thymus, spleen, immune system cells, carotid body, reproductive tract, and endothelial cells;” most of which also contain melatonin biosynthetic enzymes [31]. Dysfunction in melatonin production or release have been associated with pathologic states involving the nervous system, such as Alzheimer’s disease and Parkinson’s disease. Reduced melatonin has also been implicated in dermatologic, psychiatric, cardiovascular, and genitourinary disorders including atopic dermatitis, depression, myocardial infarction, vasculitides, erectile dysfunction, and numerous cancers. Melatonin has been investigated as potential treatment for a myriad of pathological conditions ranging across organ systems.

Many different issues can lead to sleep or circadian rhythm disorders. Any pathology or mutation that interferes with secretion or stimulation for secretion of melatonin can also lead to these disorders. Sleep disorders are conditions in which people cannot get adequate sleep, either from duration or quality [44]. About 20% of the US population suffers from one of the approximately 80 described sleep disorders, including insomnia, sleep apnea, restless legs syndrome, narcolepsy, and circadian rhythm disorders [44]. Circadian rhythm disorders include delayed sleep phase disorder, advanced sleep phase disorder, jet lag, shift work disorder, irregular sleep-wake rhythm, and non-24-h sleep-wake syndrome [45]. Symptoms of these disorders include insomnia, excessive daytime sleepiness, difficulty waking up in the morning, sleep loss, depression, poor work or school performance, and inability to meet social obligations [45].

## 4. Results

After thorough analysis of the selected papers, there are several themes and gaps that emerge from the literature. These themes can be divided into experimental variables or study design, disease or pathologic states, and treatments. The results from the literature search are shown below in Table 1. Important study characteristics and variables are displayed below as well as a brief summary of the study findings. 

## 5. Discussion

Melatonin can be measured in a variety of ways, including salivary melatonin, serum melatonin or urinary 6-Hydroxymelatonin, a melatonin metabolite. Salivary detection was utilized in the vast majority of studies, with urinary and serum detection garnering use in only a handful of papers. The overall impression is that salivary collection appears to be a more convenient and cost-effective option as only a cotton swab, as opposed to the phlebotomy necessary for serum collection or urination into a cup, is required. Additionally, in the cases of sleep studies, patients need not be awakened for a saliva collection, as Ogłodek et al. utilize passive collection while subjects slept. In 2018, Cipolla-Neto et al. stated that the gold standard for measurement is either melatonin in the serum or saliva, or urine 6-sulfatoxymelatonin during a morning collection [65]. Historically, Nowak et al. stated that both salivary and urinary melatonin measurement methods were strongly correlated with serum concentrations, and would be useful as non-invasive tests for melatonin concentration [66]. More recently in 2020, Rzepka-Migut and Paprocka detailed melatonin measurement considerations in an extensive systematic review, supporting this concept [67]. They found that urinary and salivary melatonin were useful for assessing serum concentrations non-invasively; however, there are a few caveats that must be considered when collecting these data [67]. For urinary collection, liver and kidney function must be considered as melatonin is metabolized in the liver and excreted via urine [67]. Previous research has shown that when 6-sulfatoxymelatonin levels are corrected for creatinine, they strongly correlate with serum melatonin levels [67]. Salivary melatonin appears to be both temporally and quantitatively correlated with serum melatonin levels; the main consideration Rzepka-Migut and Paprocka stipulate is that salivary levels are approximately three times less than that of serum levels [67]. Because melatonin is cyclically released as part of a circadian rhythm, the timing of collection plays a significant role in the validity of the measurement. Most studies included the time of collection in their data, and many others determined dim light melatonin onset. This involves measuring melatonin levels at specified intervals throughout a 24-h period to determine the rhythm of melatonin release as well as the peak concentration. We believe that these measurement parameters lend more credence to their results.

The sleep studies included in this review were organized in one of three ways: entirely at a sleep center or laboratory setting (*n* = 9), entirely at home (*n* = 25), or sleep at home/measure in center (*n* = 15). Subjects are under additional environmental and social stresses while in a study setting during polysomnography or other sleep-related investigations. In-home studies allow subjects the comfort of sleeping in their own beds yet may sacrifice the accuracy of the equipment available in sleep center studies. Overall, study location is an important aspect of any study design. Location can potentially alter sleep results as it is heavily influenced by several factors, including stress. However, a major benefit of a tightly controlled setting is the reliability of data collection. 

Data collection, especially self-reporting, is an aspect of study design that could potentially alter results. The accuracy of self-reported data is less than that of measured and recorded data due to error in human memory, measurement bias, and potential subject dishonesty. Many of the studies reviewed utilized wrist actigraphy (*n* = 20) as a measure of circadian rhythms. The accuracy of these devices varies between brands, with many commercial products having either high or low thresholds for movement. Regardless of the accuracy of these devices, variations in patient movement during sleep may also skew data. The incorporation of time of day or night in with these measurements is paramount to understanding the true circadian rhythm occurring in the individual. An additional study design consideration is study size. Large scale trials involving melatonin as a treatment modality were not included and do not seem to have been conducted during the timeframe for this review. This review found only case reports of melatonin being used as treatment and therefore begs the question of why melatonin is not being evaluated as a potential therapy for all sufferers of sleep and circadian disorders.

Race and ethnicity are not discussed much within the papers reviewed, as there are only two papers [49,63]. It is possible that racial difference in melatonin metabolism exist but have not been explored thoroughly. Males and females were not equally represented within the papers examined. There were nine papers that looked at melatonin in the context of depression, and males with depression were not represented in the studies to the same extent as women. This could be because women suffer more from depression than men, or perhaps their physiologies are different. 

Lastly, subjects older than 65 years old were not well represented within the studies reviewed. This was an interesting discovery as this demographic is known to experience sleep and circadian rhythm issues at a much more frequent rate than those who are younger. We see this in studies of sleep latency as well as polysomnography studies and EEG studies. As we age, the less time we spend in stage 3 and stage 4 sleep, which correlates with less restful or productive sleep. Continued research that investigates the use of melatonin as a potential therapy in these individuals is necessary as it might restore or rescue sleep and circadian rhythms. 

As for themes found in disease and physiologic states, pregnancy, pre-existing disorders, and the difference between DSPD in DSWPD come into play. DSPD and DSWPD are referred to almost interchangeably in the literature. DSPD is an acronym for delayed sleep phase disorder, whereas DSWPD is an acronym for delayed sleep wake phase disorder. During pregnancy, many physiologic changes happen in the female body. It stands to reason that melatonin circadian rhythm may also be altered during this 40-week interval. Even if the rhythm is not altered, it may be more susceptible to disruption then at any other time in a female’s life. Shimada et al., demonstrated that women with health conditions during pregnancy had lower levels of melatonin secretion during the day [52]. More data are needed to support this assertion. 

Many studies focused on circadian rhythms in subjects with pre-existing conditions or disorders, and there appears to be evidence of separate pathologies affecting circadian rhythms and related melatonin rhythms. Numerous psychiatric and psychological disorders, such as autism spectrum disorder, attention deficit hyperactivity disorder, and major depressive disorder, were represented in a large proportion of the reviewed studies. Anatomical disruption to the melatonin pathway was also found to disrupt circadian rhythms, particularly pineal cysts [53]. Metabolic disorders such as gestational diabetes and obesity have been correlated with changes in the circadian rhythm [52]. Neurologic disorders, such as traumatic brain injury, coma, and hypnic headaches have also been correlated with rhythm disruption [58,59,60]. The mechanisms for the disruption of melatonin rhythms with these disorders are unclear; further research is required to further elucidate the relationship between diseases and their effect on circadian timing and rhythms. There were 14 studies that dealt with subjects with controlled disease, whereas 9 dealt with subjects with uncontrolled disease. Both of these groups were shown to have melatonin rhythms that differed from the expected. Further research is necessary to establish whether rhythms are equally disrupted in controlled and uncontrolled disease or if there is some significant difference. 

As for treatment of sleep and circadian rhythm disorders, the literature yields two main categories both modalities: light therapy and exogenous melatonin supplementation. Light, of the two aforementioned modalities, appeared much more frequently in the search then did exogenous melatonin supplementation. Artificial light has been shown to be able to entrain the human circadian rhythm. The term for this treatment is bright light therapy. Arendt and Middleton, a team of scientists working in the Antarctic circle, utilized bright light therapy to diminish psychological symptoms of disrupted rhythms such as depression, fatigue, and decreased libido [56]. The therapy shows much promise; however, more investigation is needed to prove its effectiveness in a variety of clinical scenarios. The adverse effect of a bright light therapy “overdose” can be observed throughout the world today in the form of light-emitting electronics. However, this adverse effect can be mitigated by blocking light from the high energy (blue) end of the spectrum. Many manufacturers of electronics have introduced accessibility settings that enable users to shift the spectrum of light displayed at certain times of the day, which allows for the reduction in exposure to blue light and therefore may aid reducing disruption in circadian rhythms. Blue light blocking glasses appear to be another option in mitigating exposure to blue light. More investigation into these two modalities of blue light censorship is needed in order to show their effectiveness.

Out of the papers reviewed, only 6 papers investigated the use of exogeneous melatonin as a therapy. However, in most of these studies, treatment with exogenous melatonin was able to adequately control or restore wild-type phenotype to subjects. Solaiman and Agrawal detailed the case of a patient with non-24-h sleep wake phase disorder who was given exogeneous melatonin resulting in control of his disease and reestablishment of circadian rhythms [57]. Zuculo et al. detailed a case where, following treatment, a patient with autism was able to achieve improved social functioning and increased regularity of circadian rhythms [58]. Carriere et al. studied patients with obstructive sleep apnea and gave melatonin however there was no real endpoint [60]. Lastly, Ferri et al. treated a patient with a pineal cyst with exogenous melatonin which resulted in a realignment of the disturbed circadian rhythm [53]. Other treatment arenas were also investigated. In patients tapering off of benzodiazepines, melatonin was shown to stabilize the circadian rhythm throughout the tapering process [61]. Melatonin may even be able to supplant more dangerous sleep aids, with a more favorable side effect and safety profile. A study comparing melatonin treatment to competing prescription sleep aids may be able to show this. Further investigation is warranted to discover the extent to which melatonin treatment can help with a variety of disorders, both circadian and otherwise.

## 6. Conclusions and Future Directions

Melatonin supplementation is not known to cause a classical feedback phenomenon [65], meaning that administration of the supplement should not result in tolerance or pineal atrophy. Theoretically, this should mean that melatonin can be taken indefinitely; however, practicality dictates that this must be confirmed via clinical study.

Future research should be aimed at discovering whether or not melatonin can be used to treat or is helpful in managing a wider variety of disease states. We do not know whether melatonin has equal, superior, or inferior efficacy compared to first line pharmaceuticals. We also do not have sufficient data to show that melatonin is a beneficial adjunct treatment in addition to first line pharmacotherapies. Given melatonin’s exceptional safety profile, this is worth looking into. Melatonin is not a prescription drug, meaning it can be bought over the counter and its contents and quality are not highly regulated. Differences in melatonin physiology should be further explored between differing demographic groups to determine if this would impact treatment of disease. 

A brief literature search of animal and in vitro studies researching melatonin and circadian rhythms in the last 5 years yielded: research into melatonin associated with abnormal metabolism in rodents and humans [62], melatonin treating cognitive and endocrine deficits in zebrafish [63], genetic defects that lead to impaired circadian rhythms in rats with Smith-Magenis syndrome [64], sleep-assisting drug candidates in Drosophila model [65], and use of melatonin to inhibit chemotherapy resistance in rats with breast cancer [66]. Studies in rodents have shown light-at-night (LAN) is linked to metabolic derangements, possibly due to changes in circadian rhythms and circulating melatonin. A current area of investigation includes the effect of dietary and environmental exposures on our metabolism.

The difference between acute and chronic disorders and their treatment varies based on the disease examined. For psychiatric disorders, including sleep and circadian rhythm disorders, acute can refer to 30 days, six months, or one year. Better definitions will likely be developed if use of melatonin as a treatment modality increases in popularity in a clinical setting.

The DSM-V criteria for sleep and circadian disorders differs between disorders in the determination and classification of acute and chronic disease [67]. The disorders in this category follow the acute/subacute/persistent, episodic/persistent/recurrent, or a more specific classification (Table 2).

In the DSM-V, each circadian rhythm disorder fits under the umbrella of a circadian rhythm disorder with the individual disorders classified as subtypes. The subtypes are all classified via the episodic/persistent/recurrent paradigm. The studies dealing with these disorders do not specify whether their subjects are episodic, persistent, or recurrent cases. These distinctions should be made, as they may result in different treatment methods or dosing. Investigating efficacy of melatonin as a treatment for these disorders will require knowing how the treatment affects and interacts with each subtype of the disorder. 

## Figures and Tables

**Table 1:** Literature Search Results.

Author	Number of Participants	Treatment	Measurement	Results
Allega et al., 2018 [45]	Total *N* = 103 MDD = 32 BD = 21 HC = 40	No Treatment	Activity, 6-sulphatoxymelatonin (urine)	Total BRIAN score correlated with wake after sleep onset, total activity count during sleep, and urinary 6-sulphatoxymelatonin. BRIAN
Baandrup et al., 2016 [46]	Total *N* = 48 PRM = 20 Placebo = 28	2 mg PRM or Placebo	Active-rest cycle	Melatonin can aid disrupted circadian cycle caused by benzodiazepine withdraw
Baker et al., 2017 [47]	ASD Only = 16 ASD with comorbidities = 12 Controls = 32	No treatment	Activity, salivary melatonin	Lower mean melatonin lower in the ASD with comorbidities
Bock et al., 2016 [48]	*N* = 67	No treatment	Pharmacotherapy in pediatric insomnia	Use of over-the-counter medicine and prescription have been recommended, however only 20% have formal training in pediatric sleep disorders
Bradley et al., 2017 [49]	BD = 46 Control = 42	No treatment	Sleep, melatonin, mood	50% of BD patients had abnormal sleep and lower melatonin secretion levels
Buber et al., 2016 [50]	ADHD = 27 Control = 28	No treatment	6-sulphatoxymelatonin	Melatonin production increased in ADHD patients
Burgess et al., 2016 [51]	DSWPD = 32	No treatment	Salivary melatonin	Demonstrated feasibility of at home salivary melatonin collection
Burgess et al., 2017 [52]	DSWPD = 22 Control = 18	No treatment	Salivary melatonin	Individuals with DSWPD had more variable sleep times
Burgess et al., 2019 [53]	*N* = 37	Bright light treatment	Pain, mood, sleep, circadian timing	Morning bright light treatment reduced pain intensity, post-traumatic stress disorder symptoms and increased sleep-in veterans
Carpenter et al., 2017 [54]	*N* = 50	No treatment	Salivary melatonin	Melatonin levels are related to social and occupational functioning, and timing and length of sleep
Carriere et al., 2018 [55]	*N* = 126	Various for sleep apnea	Polysomnography	Less than 18% of subjects received melatonin for sleep disorders
Coleman et al., 2019 [56]	Major depressive *N* = 8 Previous episode *N* = 9 Controls = 31	No treatment	Salivary melatonin	Actively depressed individuals had earlier melatonin onset
Crowley et al., 2016 [57]	*N* = 66 adolescents School enrollment = 34 Summer enrollment = 32	No treatment	Salivary melatonin	Development of a method to determine circadian phase in older adolescents
Danielsson et al., 2018 [58]	DSWPD = 57	Light therapy	Salivary melatonin	Daily use of the lamp assisted in prediction of sleep onset and offset
Vallim JRDS et al., 2018 [59]	*N* = 17	No treatment	Urine 6-sulfatoxymelatonin	Alterations in the circadian rhythm of patients with Fabry diseases
Bumb et al., 2016 [60]	ADHD = 74 Controls = 86	No treatment	Pineal gland volume	Pineal gland volume smaller in unmedicated ADHD patients
Esaki et al., 2016 [61]	DSWPD = 9	Amber glasses	Activity, salivary melatonin	Use of amber glasses advanced melatonin and sleep onset
Fargason et al., 2017 [62]	ADHD = 16	Bright light therapy	Salivary melatonin	Bright light therapy advanced melatonin onset, and decreased ADHD symptoms
Ferri et al., 2017 [63]	*N* = 1	Melatonin	Activity, serum melatonin	Treatment with melatonin realigned sleep-wake rhythm
Flynn-Evans et al., 2016 [64]	*N* = 127 blind women	No treatment	Urine 6-sulfatoxymelatonin	Developed a potential treatment model
Fukuda et al., 2018 [65]	*N* = 28 (male = 22, female = 6)	500 mg L-ornithine or placebo	Salivary melatonin, sleep quality	Melatonin onset delayed after ingestion of L-ornithine
Ghaziuddin et al., 2019 [66]	High Risk BD = 6 Low Risk BP = 6	No treatment	Salivary melatonin	High risk BD had earlier melatonin onset, and Low Risk BD spent more time in deep sleep
Gobert et al., 2019 [67]	Persistent Disorder of Consciousness = 2	No treatment	Urine 6- sulfatoxymelatonin	Circadian timing system was functional, and in sync with the environmental light-dark cycle
Hamers et al., 2017 [68]	10,000 lux = 57 <499 lux = 57 Standard care = 57	Bright light treatment	Salivary melatonin, cortisol in scalp	Inconclusive
Lovato et al., 2016 [69]	DSWPD = 24	No treatment	Body temperature, salivary melatonin	Self-reported sleep timing may predict therapeutically relevant circadian phase
McGlashan et al., 2018 [70]	*N* = 12	Citalopram or placebo	Salivary melatonin	Citalopram had an effect on melatonin suppression response to light, including a 47% increase in suppression observed after an acute dose of citalopram
McGlashan et al., 2019 [71]	Depression = 16 Controls = 31	No treatment	Salivary melatonin	Patients with current depression had lower levels of melatonin suppression to light
Micic et al., 2016 [72]	DSWPD = 36 (17 male, 9 female)	No treatment	Core temperature, salivary melatonin	DSWPD patients have significantly longer melatonin rhythm and temperature taus
Micic et al., 2017 [73]	DSWPD = 16 N24SWD = 3 Control = 14	No treatment	Personality factors, Sleep/wake cycle, salivary melatonin	Compared to controls, DSWPD patients had nigher neuroticism, lower extraversion, conscientiousness and agreeableness.
Miyata et al., 2016 [74]	XPA = 8 Controls = 8	No treatment	Urine 6- sulfatoxymelatonin, DNA damage markers	XPA patients had a lower peak melatonin, increase in DNA damage markers
Moderie et al., 2017 [75]	*N* = 14	No treatment	Salivary melatonin	Melatonin onset was delayed and increase in sleepiness in those with a delayed bedtime
Murray et al., 2017 [76]	*N* = 182	No treatment	Sleep diary, activity, salivary melatonin	Melatonin onset occurred later in individuals with circadian DSWPD, along with increased odds of mild depressive symptoms
Murray et al., 2019 [77]	DSWPD = 20 Controls = 16	No treatment	Salivary melatonin	DSWPD patients had reduced reaction times, greater response speed variability in the morning
Naegel et al., 2017 [78]	Hypnic headache = 9 Control = 9	No treatment	Serum melatonin at specific times, and headache attacks	No difference in melatonin secretions between headache and control patients
Oglodek et al., 2016 [79]	Severely depressed = 40 Moderate depression = 40 Mild depression = 40 Control = 40	No treatment	Salivary melatonin	Highest melatonin at 3:00AM in severely depressed females, but lower in patients with mild and moderate depression
Parry et al., 2019 [80]	Antepartum = 26 Postpartum = 24	Early night wake therapy vs. late-night wake therapy	Plasma melatonin, mood	Early wake time improved mood in antepartum, late wake time improved mood more in postpartum individuals
Robillard et al., 2018 [81]	Unipolar depressive = 35 Healthy controls = 15	No treatment	Salivary melatonin, body temperature	Delayed circadian rhythm found in 40% of individuals with depressive disorder
Santos et al., 2018 [82]	Cerebral Palsy = 33	No treatment	Salivary melatonin	Cerebral Palsy patients had higher diurnal and lower nocturnal melatonin
Shimada et al., 2016 [83]	Pregnant women with pregnancy-related complications = 58	No treatment	Salivary melatonin	Pregnant women with hypertensive or glucose disorder complications had lower melatonin secretion through the day
Slyepchenko et al., 2019 [84]	Total *N* = 111 MDD = 38 BD = 33 Controls = 40	No Treatment	6-sulfatoxymelatonin	Levels of 6-sulfatoxymelatonin were lower in BD patients
Solaiman and Agrawal 2018 [85]	Total *N* = 111 MDD = 38 BD = 33 Controls = 40	No Treatment	6-sulfatoxymelatonin	Individuals with DSWPD have more irregular sleep, caused by timing of sleep relative to circadian phase
Solheim et al., 2019 [86]	N24SWD = 1	3 mg melatonin	Sleep diary	Sleep disorder under adequate control
Maria et al., 2017 [87]	MDD = 30	20–40 mg of fluoxetine	Salivary melatonin	Advancement of melatonin onset relative to sleep is associated with more severe depression symptoms in men, where for women a shorter window is associated with more severe depression symptoms after 2 weeks of fluoxetine
Swanson et al., 2020 [88]	Alcohol use disorder, no liver disease = 20 Control day workers = 11 Control night workers = 11	0.5 g of alcohol/kg body weight for 7 days after work, prior to bedtime	Plasma melatonin, rest-activity rhythm	Chronic and moderate consumption of alcohol for 1 week disrupted circadian rhythm
Van der Maren et al., 2018 [89]	*N* = 28 Delayed group = 14 Non-delayed group = 14	No treatment	Salivary melatonin	In delayed group, there was a higher exposure to white and blue light after melatonin onset
Watson et al., 2018 [90]	DSWPD = 12 Controls = 12	No treatment	Salivary melatonin, Phase shifting	Greater phase delay shift and increased light sensitivity in DSWPD patients.
Weissova et al., 2018 [91]	RBD patients = 10 Controls = 10	No treatment	EEG, EOG, EMG, ECG, circadian rhythm analysis, Serum melatonin	Melatonin profile in RBD patients was delayed 2 h compared to controls, and dispersed melatonin range
Wilson et al., 2018 [92]	DSWPD = 12 Control = 12	No treatment	Plasma melatonin	DSWPD individuals had a later light exposure pattern
Zuculo et al., 2017 [93]	*N* = 1	3 mg melatonin	Activity, behavior	Melatonin aids in synchronizing endogenous rhythms

Abbreviations: ADHD = Attention deficit hyperactivity disorder; ASD = Autism Spectrum Disorder; BD = Bipolar Disorder; DSWPD = Delayed Sleep-Wake Phase Disorder; ECG = Electrocardiography; EEG = Electroencephalography; EMG = Electromyography; EOG = Electro-oculography; HC = Healthy Control; MDD = Major Depressive Disorder; N24SWD = Non-24-h sleep-wake disorder; PRM = Prolonged release melatonin; RBD = Rapid Eye Movement Sleep Behavior Disorder; XPA = Xeroderma pigmentosum.

**Table 2:** DSM-V Time Criteria for Sleep and Circadian Rhythm Disorders.

Sleep or Circadian Disorder	Time Criteria
Insomnia	Episodic: >1 month and <3 months; Persistent: >3 months; Recurrent: >2 episodes within the space of 1 year
Hypersomnolence Disorder	Acute: <1 month; Subacute: 1–3 months; Persistent: >3 months
Narcolepsy	3 episodes/week over 3 months
Obstructive Sleep Apnea Hypopnea	5 obstructive apneas/hour of sleep
Central Sleep Apnea	5 central apneas/hour of sleep
Sleep-related Hypoventilation	No time criteria
Circadian Rhythm Sleep-Wake Disorders (6 subtypes–Delayed Sleep Phase type, Advanced Sleep Phase type, Irregular Sleep-Wake type, Non-24-h Sleep-Wake type, Shift Work type, and Unspecified type)	Episodic: >1 month and <3 months; Persistent: >3 months; Recurrent: >2 episodes within the space of 1 year

## Data Availability

This study did not report any data.
